# Mechanisms controlling the impact of multi-year drought on mountain hydrology

**DOI:** 10.1038/s41598-017-19007-0

**Published:** 2018-01-12

**Authors:** Roger C. Bales, Michael L. Goulden, Carolyn T. Hunsaker, Martha H. Conklin, Peter C. Hartsough, Anthony T. O’Geen, Jan W. Hopmans, Mohammad Safeeq

**Affiliations:** 1 Sierra Nevada Research Institute, University of California, 5200 North Lake Road, Merced, CA 95343 USA; 2 Department of Earth System Science, University of California, Croul Hall, Irvine, CA 92697–3100 USA; 3 USDA Forest Service, Pacific Southwest Research Station, 2801 East Sierra Avenue, Fresno, CA USA; 4 Department of Land, Air, and Water Resources, University of California, One Shields Avenue, Davis, CA 95616–8627 USA

## Abstract

Mountain runoff ultimately reflects the difference between precipitation (*P*) and evapotranspiration (*ET*), as modulated by biogeophysical mechanisms that intensify or alleviate drought impacts. These modulating mechanisms are seldom measured and not fully understood. The impact of the warm 2012–15 California drought on the heavily instrumented Kings River basin provides an extraordinary opportunity to enumerate four mechanisms that controlled the impact of drought on mountain hydrology. Two mechanisms intensified the impact: (i) evaporative processes have first access to local precipitation, which decreased the fractional allocation of *P* to runoff in 2012–15 and reduced *P-ET* by 30% relative to previous years, and (ii) 2012–15 was 1 °C warmer than the previous decade, which increased *ET* relative to previous years and reduced *P-ET* by 5%. The other two mechanisms alleviated the impact: (iii) spatial heterogeneity and the continuing supply of runoff from higher elevations increased 2012–15 *P-ET* by 10% relative to that expected for a homogenous basin, and iv) drought-associated dieback and wildfire thinned the forest and decreased *ET*, which increased 2016 *P-ET* by 15%. These mechanisms are all important and may offset each other; analyses that neglect one or more will over or underestimate the impact of drought and warming on mountain runoff.

## Introduction

The hydrology across a major river basin responds to multi-year dry periods, with the annual water balance given as *Q = P* − *ET* − *ΔS*, where *Q* is basin discharge (runoff), *P* is precipitation, *ET* is evapotranspiration, and *ΔS* is the change in subsurface storage within the basin. The impact of drought on water supply (*Q*) therefore reflects the difference between *P* and (*ET* + *ΔS*). Major droughts reduce *P*, which directly reduces *Q*; but quantifying this non-linear impact is complicated by the additional effects of drought on *ET* and *ΔS*. A variety of mechanisms may alter *ET* and *ΔS* during and after drought, and potentially intensify or alleviate the impact on *Q*. These possible mech anisms include: (i) priority allocation of *P* to *ET*, as *ET* may not increase or reduce in proportion to *P*, thus shifting the fraction of local *P* partitioned to *Q* during dry periods, (ii) changes in either meteorology and evaporative demand, or vegetation structure and transpiration, that alter *ET* and either increase or decrease *Q*, and (iii) spatial heterogeneity and the covariance between *P*, *ET* and *ΔS* that shift the location and relative importance of source regions for *Q*. Analyses of the hydrologic impact of drought have often focused on the importance of *P*, and these additional mechanisms are seldom measured and remain incompletely understood^1,2^.

Quantitatively understanding the relative importance of these amplifying and mitigating mechanisms on *ET* and *Q* across mountain basins is central to regional and global water security in a warming climate. While past studies have modeled drought magnitude and mountain runoff declines in a warmer climate, they have assumed, rather than diagnosed using quantitative observations, the mechanisms that impact hydrologic response^3,4^. Past studies have projected temperature effects of warming on mountain evapotranspiration based on both measurements and modeling, yet have not explicitly linked these changes with regolith water storage and multi-year drought^5,6^.

**Figure 1:**
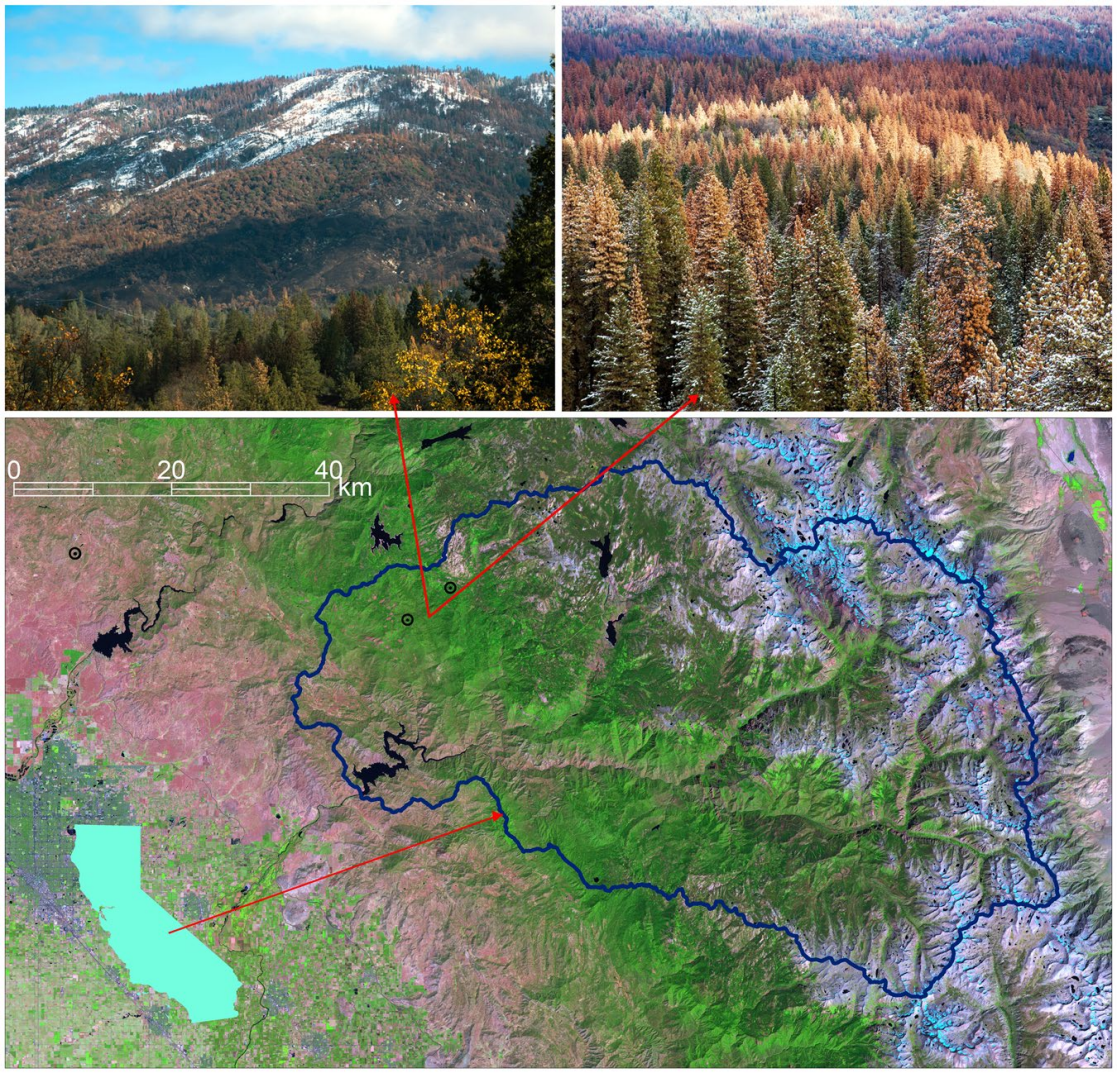
Location map. Background is false color Landsat 5 Thematic Mapper image for September 4, 2011 (red is band 5, green is band 4, blue is band 3), the year prior to the drought. The blue polygon is the Upper Kings River watershed, above Pine Flat Reservoir. The city of Fresno is at the southwestern corner of the image. Black circles are the 3 focal measurement sites, right to left: San Joaquin, Soaproot Saddle, Providence Creek. Insert in lower left shows location of the Kings River basin within California. Photos show recent tree mortality in southern Sierra Nevada Pine-Oak and mixed-conifer forests, in the vicinity of Soaproot Saddle. Photo: Margot Wholey, Dec 17, 2015.

The 2012–15 California drought provides an extraordinary opportunity to enumerate these mechanisms. The mean precipitation in California’s southern Sierra Nevada was about 50% of average for the 2012–15 water years (water year begins prior Oct. 1). While California has experienced ten multi-year below-normal precipitation periods in the last 100 years, the period that began in fall 2011 was especially severe owing to higher temperatures than in past dry periods^7–9^. This led to declining summer streamflow and widespread tree mortality, especially in the Sierra Nevada-Central Valley region (Fig. 1). The California drought, in combination with a rich suite of observations at the Southern Sierra Critical Zone Observatory (SSCZO), provide an excellent opportunity to close the water balance and evaluate the implications of subsurface water storage (represented by *ΔS*), vegetation feedbacks and spatial heterogeneity for the hydrologic mechanisms.

## Water-balance and subsurface-storage measurements along an elevation gradient

A wide suite of spatially distributed measurements of evapotranspiration, precipitation as rain and snow, snowpack and soil-water storage and stream runoff were installed in the SSCZO well before the 2012–15 drought and continue through present (Fig. 1)^10^. These include field sites along the major axis of variation in climate and hydrology, the steep elevation and climate gradient of the southern Sierra Nevada^11^. These sites were placed to both characterize key elevation zones and allow broader regional scaling. During the 2012–15 drought we observed significant changes to the water balance at three intensive-measurement sites, findings that were consistent with changes observed throughout the 3989 km^2^ Kings River basin (275–4250 m elevation).

**Figure 2:**
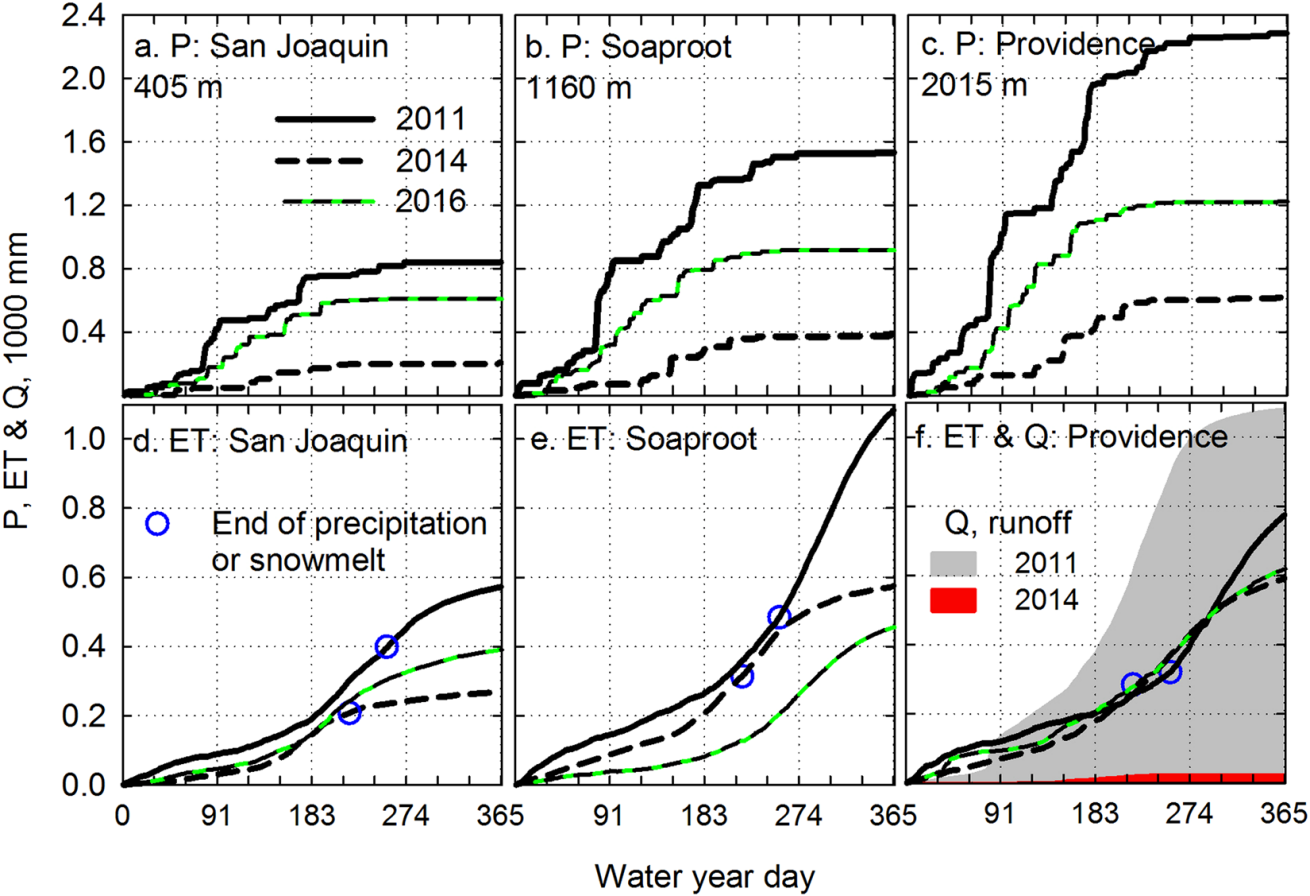
Cumulative daily precipitation (P) (panels **a–c**) and evapotranspiration (ET) (panels **d–f**) for 2011, 2014 and 2016, for 3 measurement sites: San Joaquin (oak savannah), Soaproot (pine-oak forest) and Providence (mixed-conifer forest). Runoff (Q) for one catchment at Providence also shown on panel (**f**). ET measured by eddy correlation. Circles on lower panels indicate last day of precipitation and snowmelt for 2011 and 2014, marking the day after which all evapotranspiration and streamflow came from storage that was not replenished until the next rain (right after end of water year. See Figure S1 for other years.

The three focal measurement sites represent an approximate three-fold precipitation increase along a 1600-m elevation gradient (Fig. 2a–c). Annual precipitation in water-year 2014, the 3^rd^ dry year, was about 26% of that in 2011, the wet year just before the drought (Table 1), and 50% of the 2009–2016 mean. For the same years, annual *ET* measured at the San Joaquin (oak savannah, 405 m elevation), Soaproot (pine-oak forest, 1160 m) and Providence (mixed-conifer forest, 2015 m) sites dropped 52, 47 and 20%, respectively (Fig. 2d–f).

**Table 1:** Water-balance summary for water years 2011 and 2014, in mm^a^.

Location	Annual precipitation		Evapotranspiration				Annual runoff^c^	
			Annual		Post rain or melt^b^			
	2011	2014	2011	2014	2011	2014	2011	2014
San Joaquin	839	205	575	274	175	65	—	—
Soaproot	1532	394	1098	577	598	264	—	—
Providence	2283	646	777	617	454	333	1078	27

^a^See Fig. S1 for other years. ^b^See Fig. 2 for period of rain or snowmelt. After June 12 in 2011 and May 7 in 2014. ^c^Only measured at Providence. Amounts post rain or melt were 184 and 4 mm for 2011 and 2014, respectively. Based on periodic visits over the past 8 years, streamflow at Soaproot and San Joaquin was mainly limited to periods during and shortly after rainfall.

The seasonal patterns of evapotranspiration in this Mediterranean climate shifted in response to changes in water availability. *ET* during wet years at San Joaquin was greatest in March through June, and declined by July with grass senescence (Fig. 2d). San Joaquin *ET* during the drought years declined in April. Wet-year *ET* at Soaproot increased in April and remained high through September. Dry-year *ET* at Soaproot dropped off by June, about one month after precipitation ended (Fig. 2e). Wet-year *ET* at Providence increased in June, near the end of snowmelt and two months later than at Soaproot (Fig. 2f). *ET* at Providence was comparatively consistent between wet and dry years, with a modest decline during the last two months of water-year 2014.

**Figure 3:**
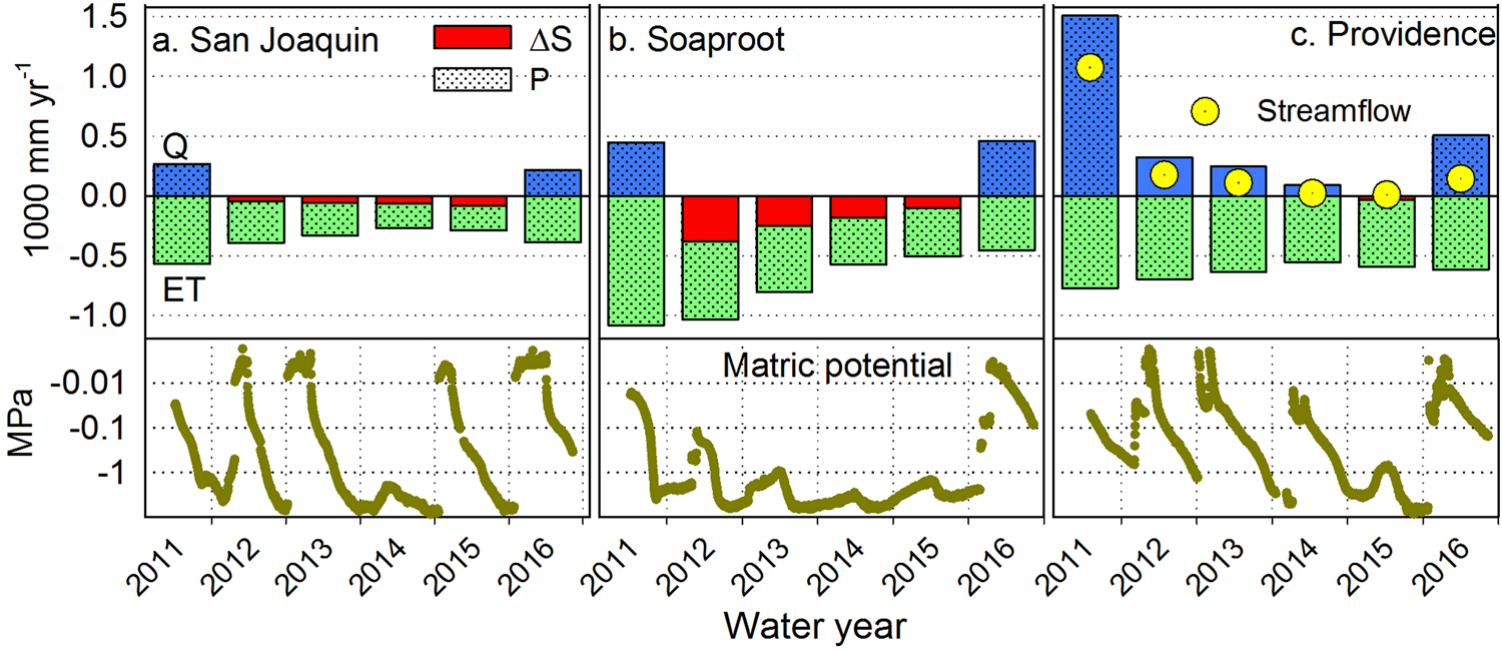
Water balance based on measured precipitation and evapotranspiration (from Figs 2 and S2) for (**a**) San Joaquin (oak savannah), (**b**) Soaproot (pine-oak forest) and (**c**) Providence (mixed-conifer forest). Height of bar above zero line indicates runoff (*Q*) (estimated as *P-ET*, if positive), height below zero line indicates evapotranspiration (*ET*), stippled area indicates annual precipitation (*P*) and red shaded area indicates amount of annual evapotranspiration coming from regolith storage (*ΔS*), calculated as *ET* in excess of annual *P*. Measured streamflow at Providence shown for comparison (from Figure S2). Lower panels show matric potential at 2-m depth for the same sites.

The seasonal patterns of evapotranspiration reflect the withdrawal and depletion of subsurface moisture that was recharged earlier in that year. We identified the dry season as the period from the last day of rain or snowmelt until the onset of precipitation, and used this interval to calculate the cumulative dry-season evapotranspiration. The 2011 cumulative dry-season *ET* at San Joaquin and Soaproot was 175 and 598 mm yr^−1^, respectively, underscoring the importance of deep rooting and the large supply of subsurface moisture during wet years that supports dry-season gas exchange. The cumulative dry-season *ET* declined by 50% as the drought progressed, coincident with a decline in subsurface moisture (Figs 2 and 3 and Table 1).

Considering a wet-year evapotranspiration of 600 mm yr^−1^ and an estimated average annual precipitation of just over 500 mm yr^−1^, evapotranspiration in the oak savannah (San Joaquin) responds to precipitation on a year-to-year basis, with subsurface storage providing little inter-annual buffer (Fig. 3a). Pre-drought (2011) *ET* in the pine/oak forest (Soaproot) was nearly 1100 mm yr^−1^, versus a pre-drought estimated 3-year (water years 2009–11) annual mean precipitation of just over 1300 mm yr^−1^ (Fig. S1). The estimated 598 mm yr^−1^ of subsurface storage was apparently sufficient to sustain a high *ET* by the forest during the dry 2012 water year, but not 2013–15, as indicated by declining *ET* (Fig. 3b).

At Providence, cumulative dry-season evapotranspiration was 454 mm yr^−1^ in 2011, and dropped about 24% in 2014 (Table 1). Measured annual runoff dropped from over 1000 mm yr^−1^ in 2011 to near zero in 2014 for a headwater catchment at Providence. Adding measured baseflow amounts for the days after the end of precipitation and snowmelt to those evapotranspiration values gives total withdrawal from storage for Providence of 638 and 338 mm yr^−1^ for 2011 and 2014, respectively.

Soil matric-potential measurements at Soaproot point to a failure to recharge at the 2-m depth beginning in 2012, compared with annual recharge for most years at Providence (Fig. 3e,f). This multi-year decline at Soaproot is consistent with the large *P-ET* deficit for the site. Providence exhibited a multi-year decline in *Q* and *ET*, with a storage deficit and decrease in matric potential by 2015; and San Joaquin showed a small but growing storage deficit through the drought. These dry summer conditions also suggest that net lateral flow into the site was small.

Our estimates of at least 600 mm yr^−1^ of evapotranspiration from storage at Providence and Soaproot are within prior estimates of water-storage capacity. Weathered granodiorite rock has observed porosities over 32%^12^. A recent geophysical survey at Providence found a weathering zone ranging in thickness from 10 to 35 m^13^. Assuming a 10-m deep weathering and rooting zone, and a plant-available water-holding capacity of weathered Sierran granitic rock of between 13 and 20%, yields a usable water storage of at least 1300 mm yr^−1,^^14,15^. The 638 mm yr^−1^ of evapotranspiration and runoff coming from storage during the dry season at Providence implies that root-accessible water occurs at depths that are well below 2.5 m.

## Kings River basin water balance and storage

**Figure 4:**
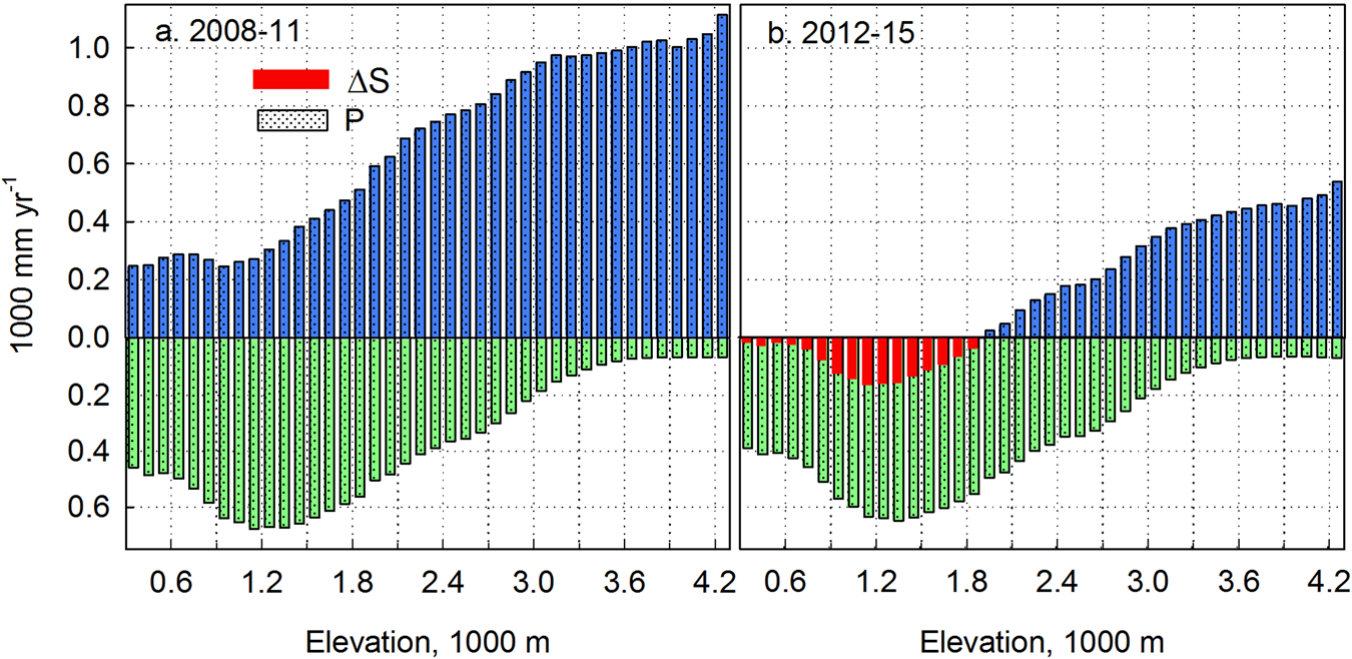
Kings River basin annual (water year, Oct-Sep) water balance by elevation for (**a**) pre-drought (2008–11) and (**b**) drought (2012–15) years. Height of bar above zero line indicates runoff (*Q*), height below zero line indicates evapotranspiration (*ET*), stippled area indicates annual precipitation (*P*) and red shaded area indicates amount of annual *ET* coming from regolith storage, calculated as *ET* in excess of annual precipitation. *ET* estimated from MODIS and *P* from PRISM data. See Figure S3 for values of *P*, *ET* and *P-ET* by year, by elevation band.

Precipitation increases with elevation and evapotranspiration peaks at mid elevation in the Kings River basin, with water limitation prevalent at lower elevation and temperature limitation higher up^6^. Annual precipitation averaged over the 4 years prior to the drought (2008–11) effectively equaled or exceeded evapotranspiration at all elevations, but was less than *ET* below about 2200 m during 2012–15 (Fig. 4). Higher elevations had sufficient precipitation and multi-year subsurface storage to both sustain vegetation and provide runoff through the dry years.

**Figure 5:**
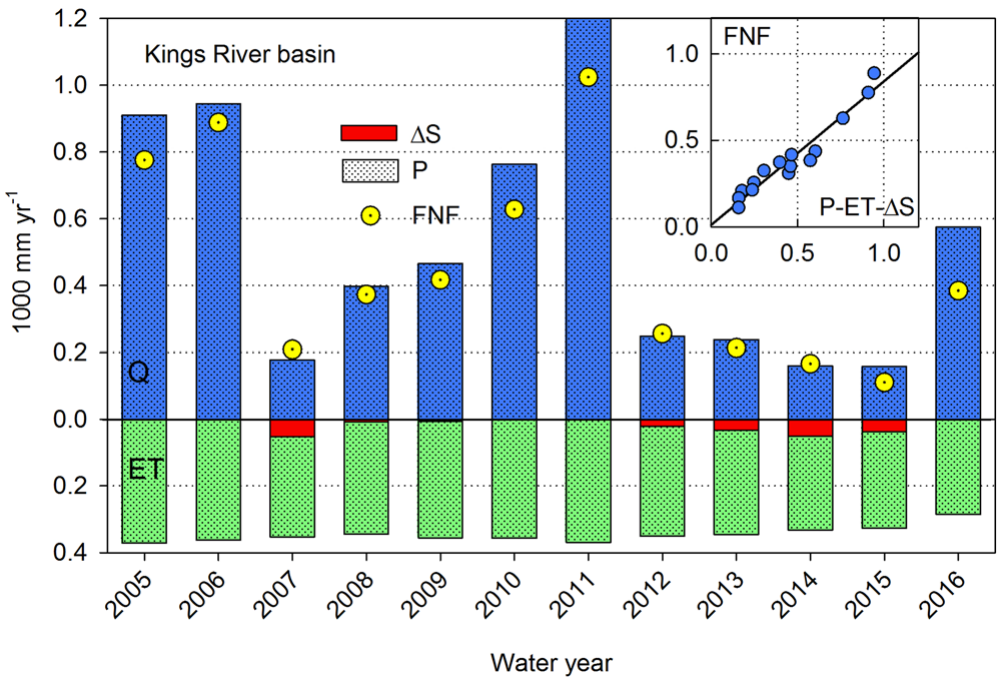
Kings River basin annual (water year, Oct-Sep) water balance by year. Height of bar above zero line indicates runoff (*Q*), height below zero line indicates evapotranspiration (*ET*), stippled area indicates annual precipitation (*P*) and red shaded area indicates amount of annual *ET* coming from regolith storage, calculated as *ET* in excess of annual precipitation at each pixel, summed over the basin. *ET* estimated from MODIS and *P* from PRISM data. See Figure S3 for values of *P*, *ET* and *P-ET* by year, by elevation band. Inset shows full-natural flow (FNF) for Kings basin versus *P-ET* for areas with water surplus (*P-ET−ΔS*), and best-fit line. Full-natural flow, the calculated runoff that would occur in the absence of human influences (dams, diversions) data from California Department of Water Resources (http://cdec.water.ca.gov/), accessed Nov 27, 2016.

Basin-wide *P-ET* (equal to *Q* + *ΔS* on Fig. 5) ranged from about 100 mm yr^−1^ in 2014 to nearly 1200 mm yr^−1^ in 2011. Summing only *P-ET* for areas with positive values corresponds well with annual full-natural flow (Fig. 5 insert). In dry years, the quantity *ΔS* reflects the basin-average *P-ET* deficit, or the amount of evapotranspiration that must be provided by a change in subsurface water storage. Full-natural-flow values higher than this calculated *Q* (equal to *P-ET−ΔS*) reflect additional withdrawals from storage. In wet years, the full-natural flow is less than *P-ET*, reflecting some of the difference replenishing subsurface storage that was depleted in dry years (Fig. 5). We note that *ΔS* is operationally defined by the water-balance equation, and is based on measurements of *P*, *ET* and *Q*. It is thus related to root-accessible subsurface storage, rather than to specific physically defined parts of the regolith.

Water years 2008–11 received near or above-average precipitation. Basin-average *ET* varied little over 2005–13, averaging 360 mm yr^−1^ (Figs 5 and S2). ET began to decline in 2014 with a widespread loss of canopy from wildfire and also tree mortality. Marked tree mortality that accelerated in summer 2015 led to a large reduction in leaf area, especially at 1000 to 2000 m elevation. The Rough Fire in summer 2015 covered 614 km^2^, with nearly all of this area falling in the Kings basin. Both of these events occurred in the fourth year of the drought and in combination led to a basin-wide *ET* decline for 2016 of about 70 mm yr^−1^ from previous years (Figs 5 and S2).

## Controls on mountain evapotranspiration and runoff during multi-year drought

Our analysis demonstrates closure of the annual water balance at multiple scales, and in doing so paints a consistent picture of the altitudinal and temporal response to drought in California’s Sierra Nevada. Our strategy relied on several independent lines of evidence, and the use of complementary *in-situ* and remotely sensed observations. The consistent patterns we observed at both the individual focal sites and across the entire basin indicate that the trends we observed are most consistent with the effect of drought.

We enumerated the four mechanisms that modulated the impact of drought on *P-ET*: (i) evaporative processes have first access to local precipitation, which increased the fraction of *P* in 2012–15 going to *ET* and reduced *P*-*ET;* (ii) 2012–15 was 1 °C warmer than during the previous decade, which increased *ET* and reduced *P-ET;* (iii) spatial heterogeneity and the continuing supply of runoff from higher elevations effectively helped to sustain *Q* in 2012–15; and (iv) drought-associated dieback and wildfire thinned the forest and decreased subsequent *ET*, which increased *P-ET* in 2016.

The first mechanism is a simple result of the priority partitioning of precipitation to evapotranspiration vs discharge (Figs 2, 3, 4 and 5). Local precipitation is initially stored in the soil and regolith (*ΔS*) and then withdrawn to support evapotranspiration. The partitioning of local *P* to *Q* is minor until the subsurface is fully saturated, at which point runoff increases markedly. The proportion of *P* allocated to *Q* therefore declines during drought, which creates a non-linear relationship between *P* and *Q*, and intensifies the impact of drought on discharge (Figs 3, 5). We estimated the magnitude of this impact by comparing the basin-wide *P*-*ET* we observed for 2012–15 with the *P*-*ET* expected if the partitioning of *P* to *Q* had remained similar to that observed during the previous decade (Fig. 5). (P-*ET*)/*P* averaged 57% for 2001–11 and 31% for 2012–15. This change in partitioning effectively decreased the *P*-*ET* for 2012–15 by 125 mm yr^−1^ across the entire basin, or 30% of the long-term average *P-ET*.

The second mechanism reflects the effects of warmer than normal conditions during 2012–15 on *ET*. Evapotranspiration varied both temporally within and spatially between the eddy-covariance towers in direct proportion to temperature and saturated vapor pressure. The area-weighted mean air temperature across the Kings River basin was 8.4 °C in 2001–11 and 9.4 °C in 2012–15, which yielded a 12% increase in saturated vapor pressure. In turn, this increase in saturated vapor pressure would be expected to have increased *ET* in 2012–15 by 12–15% and decreased *P*-*ET* by at least 25 mm yr^−1^ across the entire basin, or 5% of the long-term mean *P-ET*.

The third mechanism reflects spatial heterogeneity across the basin and the increasing importance of high-elevation source regions during the drought. Precipitation, *ΔS* and especially *ET* are heterogeneous across the Kings basin, with peak *ET* at 800–2400 m elevation. The 2012–15 drought shifted the area with a positive *P-ET* (source regions) upslope (Fig. 4), which sustained higher rates of river flow during the drought than would have occurred if the overall basin had been more homogenous. The basin-wide *ET* averaged 360 mm yr^−1^ in 2001–11 and 340 mm yr^−1^ in 2012–15. The basin-wide *P* averaged 495 mm yr^−1^ in 2012–15, and the basin-wide *P-ET* would have been 155 mm yr^−1^ in 2012–15 if the basin were homogenous. However, the basin-wide average *P-ET* considering only locations that maintained *P* > *ET* during this period and disregarding locations with *P* < *ET* was 195 mm yr^−1^ in 2012–15. In effect, these lower, negative-water-balance locations became irrelevant to discharge during the drought, which shifted the source regions upslope and maintained a basin wide *P*-*ET* that was 40 mm yr^−1^ higher than for a homogenous basin, or 10% of the long-term mean *P-ET*

The fourth mechanism reflects the effects of drought-associated leaf-area declines on subsequent *ET*. The fourth year of the drought was accompanied by widespread conifer death and also a large wildfire that affected nearly 20% of the basin. Conifer mortality was especially pronounced at the lower-elevation Soaproot site, which was originally closed-canopy pine forest. This mortality at Soaproot lead to a marked decline in *ET* in 2015, with little or no recovery in 2016 despite the return of near-average *P* (Figs 2 and S1) and in 2017 despite well above-average *P* (personal observation). The effect of mortality and wildfire below 2500-m elevation were apparent at the basin level (Figs 5 and S2). The basin-wide mean *ET* for 2001–15 was 355 mm yr^−1^ and the mean for 2016 was 285 mm yr^−1^. The mortality- and wildfire-associated thinning increased basin-wide *P*-*ET* by 70 mm yr^−1^, or 15% of the long-term mean *P-ET*. More than half of this decline was due to wildfire (Fig. S4).

All four of these mechanisms are potentially important across the Earth’s seasonally snow-covered mountains and may offset each other. In the Kings, the sum of these mechanisms was 10% of the long-term mean *P-ET*, which is comparable to or less than the effects of three of the individual mechanisms. Projections of the effect of drought on mountain runoff are therefore sensitive to the inclusion of all four mechanisms, and analyses that neglect one or more will over or under estimate the impact on both runoff and other ecosystem services.

Spatially distributed and calibrated, state-of-the-art hydrologic models already account for most of these mechanisms. Models typically allocate *P* to *ET* and *Q* following an approach that gives first priority to *ΔS* and *ET*, which should allow a realistic representation of the first mechanism, though uncertainty over soil, regolith and rooting depth and the maximum capacity to store moisture may cause errors. Models that represent *ET* as a function of vapor pressure should realistically represent the second mechanism, though uncertainty over the effect of increasing evaporative demand on stomatal closure may generate errors. Spatially distributed models should capture the third mechanism, provided they have adequate spatial resolution and a realistic representation of the spatial distribution of *P*, *ET* and *ΔS*. Many hydrologic models lack a dynamic vegetation or disturbance and recovery component, and these models will tend to overestimate the long-term impact of drought and warming on hydrology. A spatial data record of similar length to that presented here will enable more-accurate partitioning of *P* between *ET* and *ΔS*, which is not feasible using just *Q* for calibration. Moreover, this fourth mechanism is potentially the most important, depending on how long the reduced *ET* is sustained. Rates of *ET* recovery following forest dieback or wildfire are poorly known, and the 2012–15 drought may have a long-term legacy effect on Kings River flow if recovery is slow.

## Methods

Our *in-situ* measurements focused on three sites along a steep elevation transect in the Southern Sierra Critical Zone Observatory (CZO) (Fig. 1)^10,11^. The Providence Creek site (2015-m elevation) is a Sierran mixed-conifer forest with interspersed patches of montane shrubland^16,17^. The upper canopy is mostly white fir (*A*. *concolor*), ponderosa pine (*P*. *ponderosa*), black oak (Q. *kelloggii*), sugar pine (*P*. *lambertiana*), and incense cedar (*C*. *decurrens*). The Soaproot Saddle site (1160 m) is a pine-oak forest, with a ponderosa pine and oak overstory (mainly *Q*. *chrysolepsis)*. The San Joaquin Experimental Range (405 m) is an oak savannah, with deciduous and evergreen oak (*Q*. *douglasii* and *Q*. *wislizenii)*, gray pine *(P*. *sabiniana)* and annual grasses. Estimated 1970–99 climatological precipitation is 513, 805 and 1015 mm at San Joaquin, Soaproot and Providence, respectively; and respective min/max temperatures for that period are 9.3/23.5, 5.5/18.0, and 2.7/14.8°C^11^. San Joaquin and Soaproot are rain dominated, and Providence receives up to 50% of its precipitation as snow^16^. Prior to the drought, Soaproot and Providence retained high levels of greenness all year, with San Joaquin showing senescence of grasses in summer. Tree mortality was especially severe near the Soaproot intensive-measurement site. The photos in Fig. 1 shows the landscape in an east-west running valley in the 800–1500 elevation range, in December 2015. Tree mortality became apparent between July and December in 2015 in this region (personal observation).

Regional trends in soil and regolith properties are governed by a strong bioclimatic gradient across the Sierras^18^. Soils at San Joaquin are Coarse-loamy, mixed, superactive, thermic Typic Haploxerepts with loamy sand textures and depth to weathered bedrock typically occurring between 50 and 100 cm. At Soaproot, soils are Fine-loamy, mixed, semiactive, mesic Ultic Haploxeralfs with sandy-loam and sandy-clay-loam textures and depth to weathered bedrock occurring between 150 and 250 cm. Soils at Providence are Coarse-loamy, mixed, superactive, mesic Humic Dystroxerepts with Coarse-sandy-loam textures and depth to weathered bedrock occurring between 100 and 200 cm.

Portions of all three sites were actively managed during the study: parts of the Providence watershed were thinned in water-year 2012; parts of the Soaproot site were burned by prescribed ground fire in winter 2013; much of the San Joaquin site was grazed each year. This management generally avoided the areas where we had installed equipment, but nonetheless underscores the importance of a broad research strategy that relies on multiple independent lines of evidence, including *in-situ* and remotely sensed observations. Hence, management at the three focal sites could have confounded the attribution of year-to-year variability, but the suite of evidence there and across the basin indicates that the recent trends are most consistent with the effect of drought.

Daily evapotranspiration was measured at the three sites by eddy covariance^11^. Streamflow was measured at Providence and precipitation at Providence and San Joaquin^16^. Matric potential was measured down to 2 m at the three focal sites using ceramic thermal-dissipation probes (229-L sensor, Campbell Scientific Inc., Logan, UT)^19^. We combined spatially resolved estimates of precipitation and evapotranspiration to investigate the patterns of water balance.

For the Kings River basin analysis, monthly estimates of precipitation at 4-km resolution (PRISM) were downscaled using a bilinear interpolation and summed for each water year. Annual evapotranspiration at 250-m resolution was calculated from the Normalized Difference Vegetation Index (NDVI; MOD13Q1 collection 5) and PRISM maximum air temperature using an exponential regression based on 77 site years of observations at ten eddy-covariance towers across California (Figure S3)^11^. This NDVI component of the regression exploits the bidirectional interaction between canopy density and evapotranspiration; a high NDVI indicates a high Leaf Area Index (LAI) and hence a high rate of transpiration through canopy gas exchange, and a high rate of transpiration feeds back to a high LAI and NDVI through Net Primary Production (NPP). The feedback of *ET* and NPP to LAI develops over a few years in evergreen forest, and hence the NDVI measured in a given year is strongly influenced by the water balance and evapotranspiration that occurred over the last few years. The temperature aspect of the regression accounts for the increase in ET for a given LAI and NDVI that is caused by increasing vapor pressure with warmth. Our measure of evapotranspiration can be thought of as a lagging indicator of recent historic water availability, and of the amount of evapotranspiration that is needed to support a site’s LAI. *P-ET* therefore provides an especially useful measure of drought severity and whether the precipitation in a year is sufficient to support the local vegetation density. It thus has advantages over use of potential evapotranspiration (*PET*), which is insensitive to dry years; or to *P-PET*, which is offset from the actual water deficit that is driving moisture stress.

Data for this and other SSCZO studies are available through https://criticalzone.org/sierra/ and as noted on figures.

## Electronic supplementary material

Supplemental Information
