# Supplementary material for: Mechanisms controlling the impact of multi-year drought on mountain hydrology
Source: Sci Rep. 2018 Jan 12;8:690. doi: 10.1038/s41598-017-19007-0 (PMC5766567; doi:10.1038/s41598-017-19007-0)
Supplement: Supplementary file 1 — Supplemental Information [file 41598_2017_19007_MOESM1_ESM.pdf]

Supplemental Information

Mechanisms controlling the impact of multi-year drought on mountain hydrology

Roger C. Bales[1*], Michael L. Goulden[2], Carolyn T. Hunsaker[3], Martha H. Conklin[1], Peter C. Hartsough[4], Anthony T. O'Geen[4], Jan W. Hopmans[4], Mohammad Safeeq[1]

[1]Sierra Nevada Research Institute, University of California, 5200 North Lake Road, Merced, CA, 95343, USA

[2]Department of Earth System Science, University of California, Croul Hall, Irvine, CA 92697-3100 USA

[3]USDA Forest Service, Pacific Southwest Research Station, 2801 East Sierra Avenue, Fresno, CA, USA

[4]Department of Land, Air, and Water Resources, University of California, One Shields Avenue, Davis, CA 95616-8627, USA

Figure S1 shows the cumulative precipitation, evapotranspiration and streamflow data used in the analysis. Precipitation and streamflow for Providence were measured as part of the Kings River Experimental Watersheds program[1]. Precipitation for Soaproot was from a rain gauge on the Soaproot flux tower, part of the Southern Sierra Critical Zone Observatory, and the Mountain Rest RAWS (Remote Automatic Weather Station) site, blended to develop a consistent data set over the period of analysis. Precipitation for San Joaquin was from the on-site rain gauge, maintained by the Pacific Southwest Research Station (personal communication, R. Denton) and the nearby Hurley RAWS site, blended to develop a consistent data set over the period of analysis. Evapotranspiration at the 3 sites was measured as part of the Southern Sierra Critical Zone Observatory[2].

Figure S2 shows the year-by-year annual precipitation and evapotranspiration data used in the analysis. Values are averaged by 100-m elevation bins for presentation. Evapotranspiration values were scaled across the landscape using the relationship on Figure S3; and precipitation data were from PRISM (Parameter-elevation Relationships on Independent Slopes Model). Monthly PRISM values at 4-km resolution were downscaled using a bilinear interpolation and summed for each water year.

Figure S3 is adapted from an earlier analysis relating annual evapotranspiration measured by eddy correlation at 10 flux towers in California to a Normalized Difference Vegetation Index (NDVI) from MODIS (Moderate Resolution Imaging Spectrometer)[2]. The current analysis was updated with additional years of data, plus including mean saturated vapor pressure to normalize annual evapotranspiration values. Saturated vapor pressure was based on PRISM maximum air temperature, after weighting for each site's seasonality. This seasonality was determined individually for each tower site, and also for each pixel in the gridded analysis, based on the site's seasonal pattern of leaf area, after accounting for cold limitation in winter.

In 2016 about 20% of the Kings River basin experienced a large wildfire. Areas that burned had denser vegetation that the average for the remaining 80% of the basin, and thus pre-wildfire evapotranspiration (Fig. S4). However, in areas that burned, *ET* dropped about 209 mm yr^{-1} in

2016 compared to the 2001-15 mean for the same areas. The *ET* in unburned areas dropped about 29 mm yr^{-1} in 2016 compared to the 2001-15 mean for the same areas.

References

1. Hunsaker, C. T., Whitaker, T. W. & Bales, R.C. Snowmelt runoff and water yield along elevation and temperature gradients in California's southern Sierra Nevada. *J. Am. Wat. Resour. Assn.*, **48**, 667-678 (2012).
2. Goulden, M. L. *et al*. Evapotranspiration along an elevation gradient in California's Sierra Nevada. *J. Geophys. Res.*, **117** G03028 (2012).

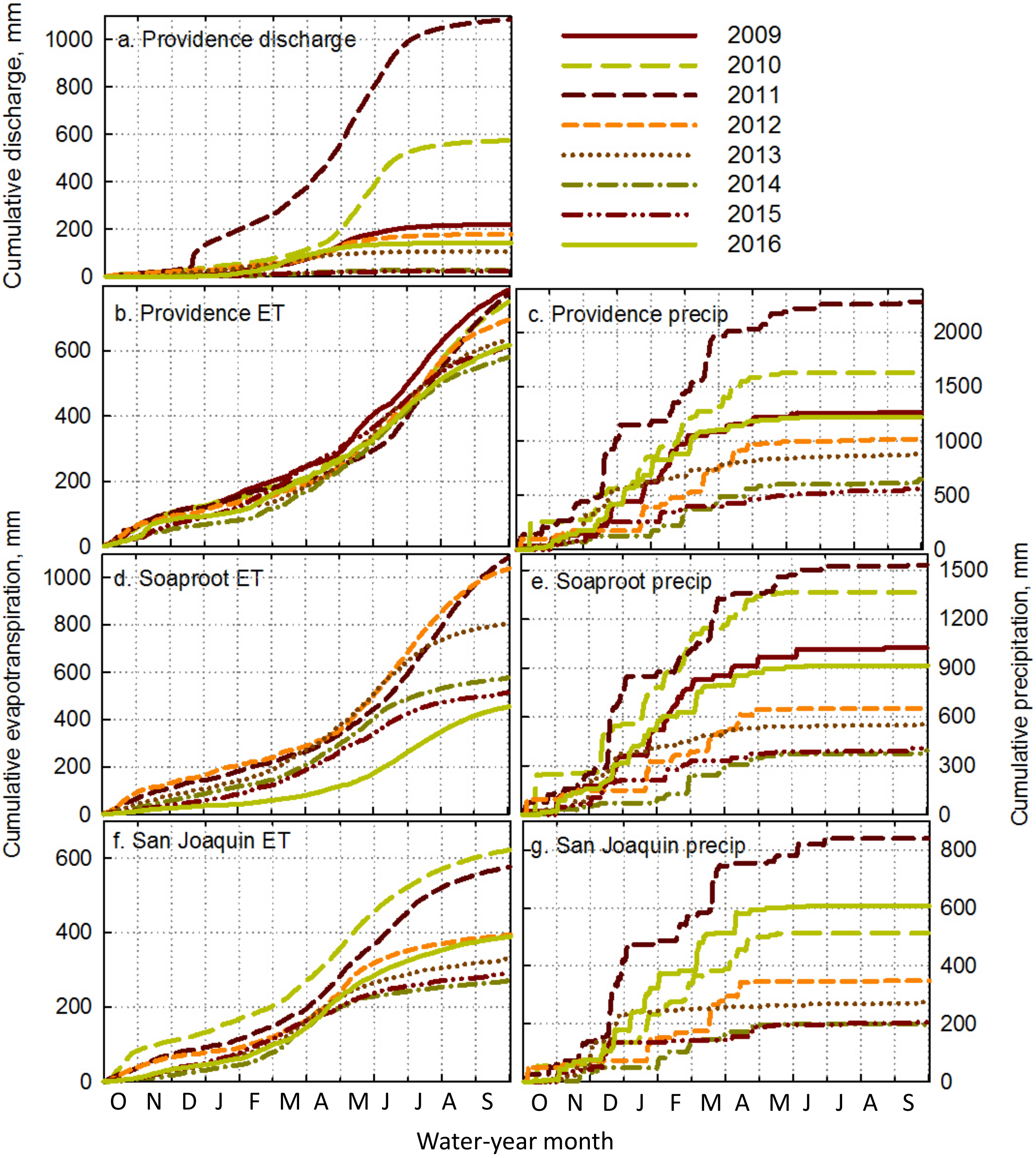

Figure S1. a) Stream discharge, b) evapotranspiration (ET) and c) precipitation for Providence, d) ET for Soaproot, e) precipitation averaged for 2 RAWS stations within 10 km of Soaproot, Fence Meadow (1514 m) and Mountain Rest (1230 m), f) ET for San Joaquin Experimental Range, and g) precipitation for San Joaquin Experimental Range. Note that for ET, only Providence has all 6 years, as the record started later at the other sites. Missing precipitation for San Joaquin for parts of water years 2011, 2014, and 2016 from Hurley RAWS station (elevation 376 m), as it had comparable precipitation values to San Joaquin for other periods.

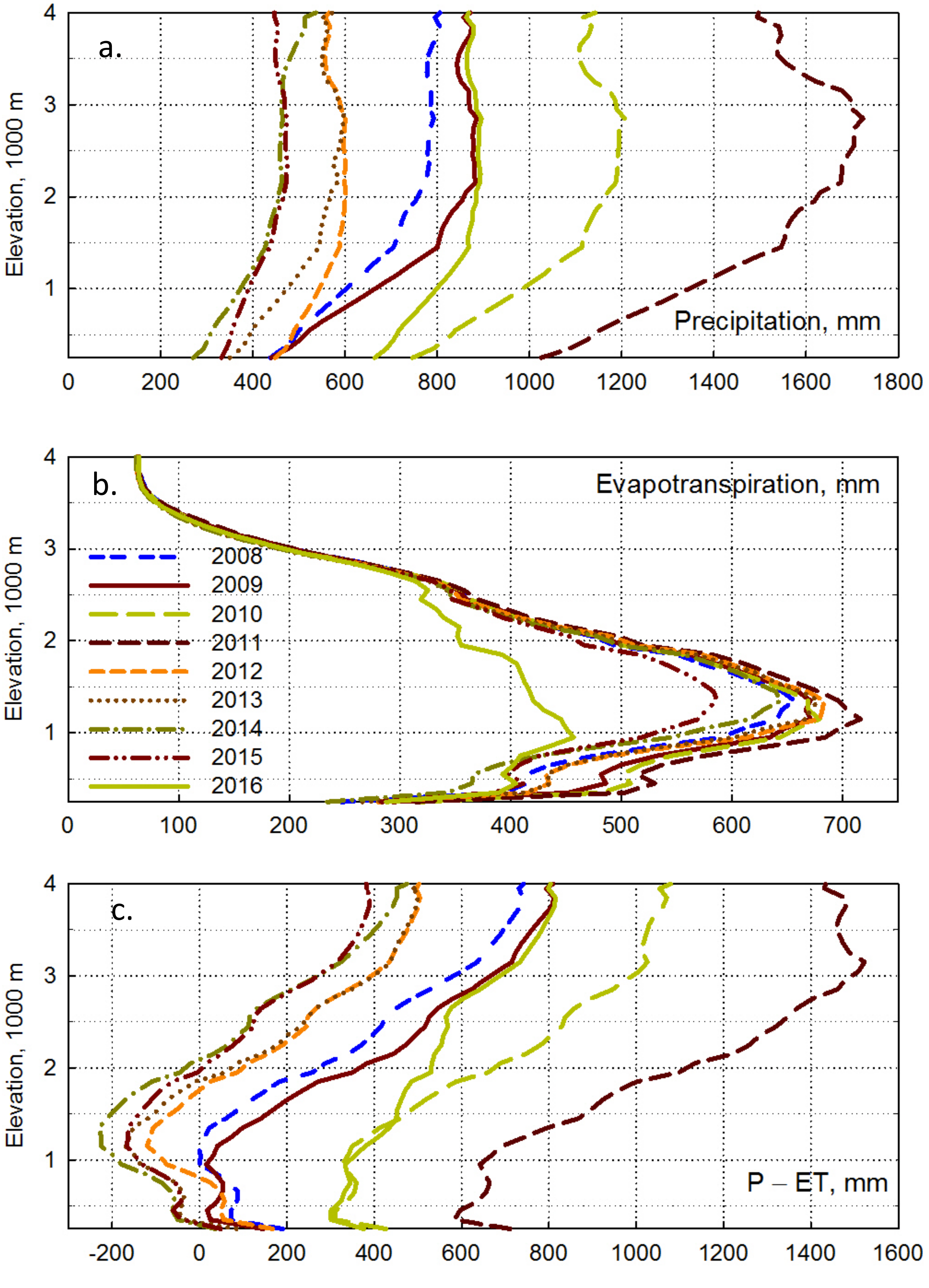

Figure S2. a) PRISM annual precipitation by water year (http://www.prism.oregonstate.edu), and b) annual evapotranspiration estimated from MODIS NDVI, and c) difference between precipitation (P) and evapotranspiration (ET). Note that the 2016 value includes the effect of the 614 km^2 Rough Fire.

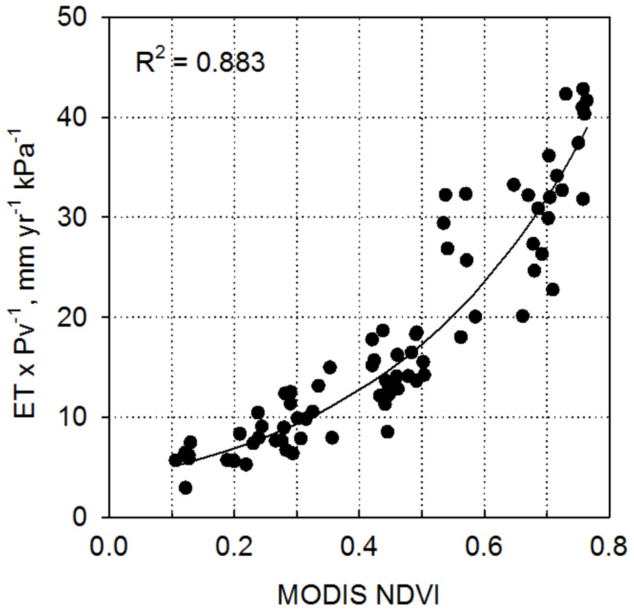

Figure S3. a) Regression used for scaling evapotranspiration (ET) across the Kings River basin, using measured annual evapotranspiration normalized by saturated vapor pressure. b) Resulting relationship between NDVI and measured ET.

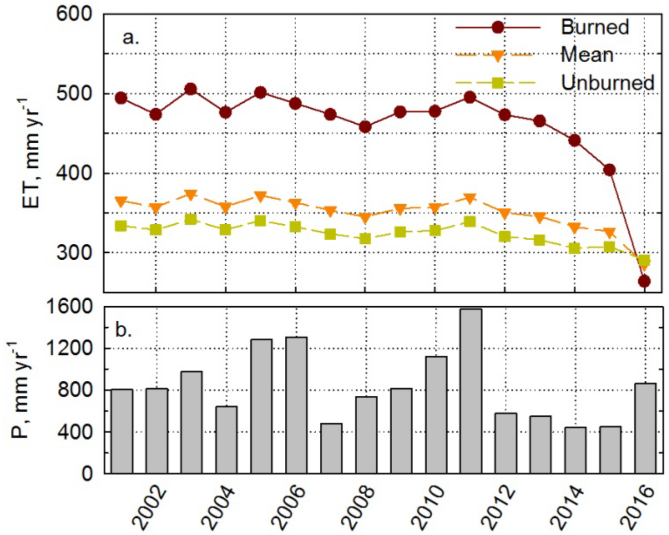

Figure S4. a) Average evapotranspiration (ET) for areas with versus without wildfire in 2016 . b) Average precipitation for the basin.
